# Differences in Squat Jump, Linear Sprint, and Change-of-Direction Performance among Youth Soccer Players According to Competitive Level

**DOI:** 10.3390/sports9110149

**Published:** 2021-10-27

**Authors:** Michael Keiner, Andreas Kapsecker, Tobias Stefer, Björn Kadlubowski, Klaus Wirth

**Affiliations:** 1 Department of Sports Science, German University of Health & Sport, 85737 Ismaning, Germany; Andreas.Kapsecker@edu.my-campus-berlin.com (A.K.); tobias.stefer@tsv1860.org (T.S.); bjoernkadlubowski@gmail.com (B.K.); 2 DSC Arminia Bielefeld e. V., 33615 Bielefeld, Germany; 3 Department of Sports Science, University of Applied Sciences Wiener Neustadt, 2700 Vienna, Austria; klaus.wirth@fhwn.ac.at

**Keywords:** talent identification, speed, football, performance diagnosis

## Abstract

The aim of this investigation was to analyze significant differences in performance depending on the level of play (elite vs. amateur) in youth soccer players (under 17 years. old (U17) and U19). A cross-sectional study was conducted, and 45 elite and amateur male youth soccer players (16.56 ± 0.9 years old) were evaluated in their performances in squat jump (SJ), 10 m linear sprint (LS), 20 m LS, 505 agility test (505) and Illinois agility test (IAT). Differences in performances were analyzed with a 2 × 2 MANOVA, post-hoc ANOVAs, and Hedges’ g (g) for pairwise comparisons of subgroups (level of play and age group). This investigation showed that the elite player performance was significantly (*p* < 0.05) better in all performance tests than amateur players in both age groups. Interestingly, this investigation showed that the more complex the target exercise, the larger the effect sizes for group differences (SJ: g = 0.64–1.18, LS: g = 0.05–2.23, change-of-direction (COD): g = 3.01–6.84). The SJ, LS, 505, and IAT may prove useful in talent selection test batteries to separate between competitive levels in youth soccer players.

## 1. Introduction

For match-play demands in soccer, players have to perform several high-intensity tasks during a game [1]. Because of changes in technical, tactical, and physical requirements, soccer matches have become more dynamic and fast paced [2,3]. It is becoming increasingly essential to consider physiological factors, such as the capacity of soccer players to produce various forceful and explosive actions, for players to exhibit optimal performance [4]. In addition to jumps, shots, and linear sprints (LS), athletes must also perform sprints with directional changes [5]. The capacity to perform change-of-direction (COD, excluding the decision-making process) is an essential physical fitness factor needed to perform effective and efficient COD maneuvers in multiple sports [6]. In modern soccer, which is speed-oriented, dynamic, and fundamentally more demanding, the physical demands of speed, especially COD speed, are of central importance for the development of the best possible performance and corresponding sports success [7,8,9]. Therefore, the ability to accelerate, change direction, and rapidly decelerate could increase the chance of players winning one-on-one duels or performing effective defending maneuvers in the match [10]. Soccer players perform approximately 727 ± 203 turns during a match [1]. A soccer player changes direction every 2–4 s and makes 1200–1400 changes of direction during a match [11]. LS, COD, and jump performances are therefore determinants of performance in soccer matches. The assessment of field-based physical performance can help coaches in evaluating the level of players, thus positively impacting the talent identification processes and training implications [12]. Since LS, COD, and jump performances also have a significant influence on the outcome of the match, it is to be expected that these performances differ between different performance levels. The literature shows performance differences between elite and sub-elite players [13] and elite and elite youth soccer players [14]. However, the literature is not exhaustive and is concordant on performance differences between elite and amateur soccer players. The literature reports small to large differences in squat jump (SJ) performance in favor of elite compared to amateur soccer players (Hedges’ g (g) = 0.3–0.97) [13,15] and 16 years old elite and youth amateur soccer players (g = 0.3) [16]. For LS (10 meters (m)), the literature shows quite large differences in favor of elite compared with amateur soccer players (g = 0.79) [13], and also for elite compared to amateur youth soccer players (12–16 years old), small to moderate differences for LS 10 m (g = 0.22–0.65) [16,17,18], large differences for 20 m (g = 0.89–0.94) [18,19], moderate to large differences for LS 30 m (g = 0.5–1.2) [20] and moderate differences for LS 40 m (g = 0.5) [21]. Comparisons between elite and amateur soccer players for distances over 30 m in LS showed moderate differences as well [13]. Only two studies analyzed different COD tests in elite youth soccer players and youth amateur players. Trajković et al. [18] found large differences in COD tests with one 45° turn (left and right direction in separated run) on a 10 m course (g = 0.8–0.95) and large differences for the Illinois test (IAT) (63–65 m, 9 turns of 90°–180°, g = 1.5) and Trecroci et al. [17] found significant differences (g = 0.69) for a COD test with six 90° turns on a 15 m course in favor of elite and regional youth soccer players (15–16 years old).

In light of the considerations above, it would be important to assess whether elite and amateur players between 16 and 19 years old are distinguishable by means of field-based physical tests or whether this difference is even more pronounced than in the studies with younger subjects [16,17,18,19,20,21]. The outcomes could provide coaches additional data on the physical performances of their under-19-year-old (U19) and under-17-year-old (U17) players to make appropriate choices in terms of talent identification at this particular age stage. From this, the research question is whether there are statistically significant differences in LS, COD, and jumping performance as a function of playing level (elite vs. amateur) in youth soccer players. It was hypothesized that performance would differ by playing level in favor of elite soccer players.

## 2. Materials and Methods

In order to answer the research question, a cross-sectional study was conducted. Forty-five elite and amateur male youth soccer players were evaluated in their performances in SJ, 10 m LS, 20 m LS, 505 agility test (505), and IAT and checked for differences in performances (elite vs. amateur players). Tests were carried out on 2 test days within a 1-week period. On test day 1, the LS was performed first, followed by the SJ. Two days later, on test day 2, the COD tests were determined. Based on the total distance in ascending order, to avoid fatiguing effects, first the 505, then the IAT test, was performed. The elite players were familiar with the IAT, 505, LS, and SJ tests because these tests were part of their semi-annual performance diagnostics routine. The amateur players do not conduct regular performance tests; therefore, one week before test day 1, the subjects completed a familiarization session on two separate days (day 1: LS, SJ; day 2: 505, IAT).

### 2.1. Subjects

Forty-five male youth soccer players (16.56 ± 0.9 (range: 15–18) years old; body mass: 70.7 ± 9.4 kg; height: 1.78 ± 0.08 m, body mass index: 22.3 ± 2.6) were recruited from U17 and U19 teams of two youth training centers. The teams of the two training centers played in different competitive levels. Within the two age groups, a comparison was made between a soccer team in the highest German junior divisions (youth Bundesliga, elite players (U17: *n* = 14; U19: *n* = 12)) and a youth soccer team in the lowest German youth league (club level, amateur players (U17: *n* = 8; U19: *n* = 11)). The soccer teams were classified elite in reference to the definition used by Lorenz et al. [22], who considered elite athletes as those who played at a higher level than peers within a sport (national (junior Bundesliga, highest league in Germany) vs. club level (district league, lowest league in Germany)). The elite players of both age groups performed 4 to 5 soccer sessions per week (1.5–2 training sessions/day), whereas the amateur soccer players of both age groups performed 1 to 2 soccer sessions per week. Both groups regularly competed in their league on weekends during the season. The subjects did not participate in fatiguing training sessions for a minimum of 3 days before testing. None of the subjects reported any injuries at the time of testing.

Each subject and their parents (if the subject was not yet 18 years old) were informed about the aims of the study and the experimental risks involved with the research. All subjects and their parents (if the subject was not yet 18 years old) provided written informed consent to participate in the present study. Furthermore, this study was approved by the Ethics Committee of the German University of Health & Sport (DHGS-EK-2021-002). The study was performed with human subjects in accordance with the Helsinki Declaration.

### 2.2. Procedures

Body mass was analyzed using a personal scale (PSD, Neuss, Germany). Body height was determined by means of a meter stick and spirit level attached to a wall. The spirit level was placed on the head of the subjects in order to be able to read the body height. The warm-up for the jump and sprint tests consisted of nonspecific running at low-to-medium intensity for approximately 5 min. Then, coordination exercises, such as running with lifted knees, heeling, and sidestepping, were performed for approximately 5 min. Subsequently, 3 acceleration runs over approximately 30 m were performed with short intervening walking breaks. Jumping performance was measured using a contact mat (Refitronic, Schmitten, Germany) that operates as a switch. This system sent information to the computer regarding whether the mat was loaded. From this information, the flight time and the jump height were determined for all jumps. The jump height was calculated from the flight time (gt^2^/8; g = the gravitational acceleration (9.81 m·s^−2^) and t = flight time). The squat jump was initiated at a knee angle of 90° without counter-movement. The subjects had 5 trials in which to achieve their best result. Between every jump, the athletes received a 1 min break. The test-retest reliability is reported to have an ICC = 0.97 [23]. The subjects performed three attempts per COD test, which were separated by a 3 min break. The best trial was used for the statistical analysis. The description of the test setups can be found in the literature [24,25]. As different COD tests have different requirements (e.g., length, turns), these tests seem to evaluate task-specific requirements [26]. Therefore, two tests that have a heterogeneous requirement profile (IAT: 63–65 m, 9 turns of 90°–180° vs. 505: 10 m, 1 turn of 180°) were selected. IAT and 505 tests were frequently used in studies with soccer players [17,18]; the choice of the same tests should lead to better comparability of own results and other studies. If the pylons or hurdle bars were knocked down or touched during COD testing, a follow-up attempt was completed. The tests were separated by a break of 15 min. LS performance was measured for a distance of 10 m and 20 m. Each athlete also had three attempts. Between each completed sprint, the athletes received a 3 min break. The time was measured for all COD and LS tests with a double-timing gate system (wk7 time watch, Ditzingen, Germany). The starting point was marked with a small cap 0.75 m away from the starting gate to avoid early triggering, e.g., by a hand movement or a bent body position. The subjects independently chose when the measurement began according to the activation of the barriers. Thus, the reaction time was excluded from the measurement. The test-retest reliability is reported to have an ICC = 0.85 for 505, ICC = 0.97 for IAT, and ICC = 0.89 for LS [23].

### 2.3. Statistical Analyses

Statistical analysis was performed using SPSS software version 27 (IBM, Ehningen, DE, Germany). Data were tested for normality using the Shapiro–Wilk test to determine whether parametric or non-parametric statistical methods were appropriate. Reliability analyses were performed using intraclass correlation coefficients (ICCs) and a 95% confidence interval (95% CI). For the reliability analyses, the ICCs and 95% CIs were calculated from the 3 trials of the testing day. ICCs greater than 0.70 indicate suitable reliability [27]. Additionally, variability was determined using mean coefficients of variation (CV %). Acceptable thresholds were determined using a CV of <10%. In addition, the variance of homogeneity or variance of heterogeneity was determined by the Levene test of equal variance. The homogeneity of covariances was assessed by Box’s test. A 2 × 2 MANOVA was calculated to show a statistically significant difference between the age group and playing level on the combined 5 dependent variables (LS 10 m, LS 20 m, SJ, IAT, 505). A second 2 × 2 MANOVA was calculated to show a statistically significant difference between the age group and playing level on combined anthropometric data (body mass, height, BMI). Furthermore, partial eta-squared values (η2) were computed for all the analyses as an indicator of the effect size. A partial eta-squared value between 0.01 and 0.06 indicates a small effect size, and a partial eta-squared between 0.07 and 0.13 indicates a medium effect size, whereas a value equal or higher than 0.14 indicates a large effect [28]. Post-hoc univariate ANOVAs were performed for each dependent variable when a significant result was observed in the MANOVA. Significant effects of ANOVA were tested by post-hoc Scheffé test for effects in the subgroups. Effect sizes Hedges’g were calculated and were defined as trivial effects between 0.01 and 0.19, as small between 0.2 and 0.49, as moderate between 0.5 and 0.79, and as large above 0.8.

## 3. Results

The Shapiro–Wilk test showed normally distributed data for variables in all subgroups and the total group. The results of the reliability analyses are displayed in Table 1. All ICCs are clearly above the limit value of 0.7 and under the limit of CV (< 10%) and can therefore be classified as suitable reliability [27].

Levene’s test calculated homogeneity of the error variances (*p* > 0.05), except for the variables body mass and BMI (*p* = 0.031, 0.005). There was heterogeneity of covariances, as assessed by Box’s test (*p* < 0.001). MANOVA showed no statistically significant difference between age groups and playing level for the combined anthropometric variables (F_(3,39)_ = 0.122, 1.786, *p* = 0.166, 0.953, partial η^2^ = 0.009, 0.121, Roy’s largest root φ = 0.009–0.137). MANOVA showed no statistically significant difference between age groups (F_(5,37)_ = 2.233, *p* = 0.071, partial η^2^ = 0.232, Roy’s largest root φ = 0.302), but between playing level (F_(5,37)_ = 81.782, *p* < 0.001, partial η^2^ = 0.917, Roy’s largest root φ = 11.052) on the combined dependent performance variables. Post-hoc univariate ANOVAs showed for every depending performance variable statistically significant results between level of play, F_(1,41)_ = 10.225–365.256, *p* < 0.003, partial η^2^ = 0.200–0.899). Table 2, Table 3 and Table 4 provide an overview of the mean values ± SD and effect sizes for subgroups of both anthropometric data (height, body mass, and BMI) and performance tests (SJ, LS and COD) in age groups and the total group. Elite player performance was significantly better in SJ, LS, and COD than amateur players. The results show that the more complex the target exercise, the larger the effect sizes for group differences (SJ: g = 0.64–1.18, LS: g = 0.05–2.23, COD: g = 3.01–6.84).

## 4. Discussion

Elite player performance was significantly better in SJ, LS, and COD than amateur players. Interestingly this investigation showed that the more complex the target exercise, the larger the effect sizes for group differences (SJ: g = 0.64–1.18, LS: g = 0.05–2.23, COD: g = 3.01–6.84).

Performance differences in favor of elite populations are basically consistent with the literature [16,17,18,19,20]. Playing soccer (and, therefore, sprinting, changing direction, and jumping) may be an effective training stimulus for improving SJ, LS, and COD [29]. The higher performance in the elite subjects might be attributed to the higher volume of playing soccer. The analysis of data in this study cannot clarify why the level of significance was not reached for LS 10 m and SJ (g = 0.64) in subgroup U17. It is possible that from a statistical perspective, the non-significant differences in the SJ of both subgroups (elite vs. amateur) in U17 can be explained by the heterogeneous performance of the subgroup amateur players. Although, the variation (CV = 4.4%) of SJ, which could be explained by the generally unusual concentric-only movement of soccer players, may have influenced the calculation. It should be noted that the two groups in the U17 age group differ with a moderate effect in SJ (g = 0.64). The non-significant results in the LS 10 m variable are contrary to the findings of performance differences depending on the playing levels of LS 10 m in U19, as well as LS 20 m in both age groups and contrary to other findings in the literature [16,18,19]. Only Trajković et al. [18] also found non-significant differences between elite and amateur soccer players in LS 10 and argued that this might be related to the maturity stage of players, which can affect sprint performance. This effect may also mask group differences in LS 10 m and SJ between the level of play in U17 in this study, as effect sizes in body height were calculated to be large (g = 1.0) in favor of the amateur players. However, a clear justification of the non-significant differences in LS 10 m and SJ between the level of play in subgroup U17 in this study cannot be found in the anthropometric data, as this study did not calculate significant differences between the subgroups (amateur vs. elite) in accordance with the literature [17].

The effect sizes of performance differences in COD tests of this study (g = 3.01–6.84) far exceeded the effect sizes found by Trecroci et al. [17] and Trajković et al. [18] (g = 0.69–1.5). It is fundamentally difficult to compare the studies, and thus also the results, with each other because different studies may have used different definitions of the subject categorization into elite, sub-elite, and amateur [17]. Here, the demand for a uniform definition of the performance level for team sports and its consistent use in the literature must be established [22]. Nevertheless, even in comparison with studies that used an identical definition of playing status and comparable or identical COD tests in terms of the requirement profile, significantly higher performance differences between elite and amateur players were confirmed in this study (g = 0.95 vs. g = 3.01–6.84) [18]. Trajković et al. [18] recruited elite, sub-elite players from national championships without naming the country of the championship. It is possible that a difference in performance between the national leagues explains the difference in effect sizes. It is also possible that the difference in the magnitude of the effects is due to the fact that the subjects measured in this study were older compared with other studies (16.56 ± 0.9 vs. 15.7 ± 0.6 years old) [18]. Here, selection factors could be used again as an explanation for larger training volumes and intensity of older elite players. Nevertheless, on the basis of the available data, it is unclear why the effect sizes differ between the studies mentioned and thus points to a further need for scientific research on this topic.

COD test performance represents the performance parameters that showed the highest effect sizes in this study (SJ: g = 0.64–1.18, LS: g = 0.05–2.23, COD: g = 3.01–6.84). Cometti et al. [13] also found higher effect sizes for performance differences depending on the level of play in linear sprint compared to jump performance; however, Trajković et al. [18] did not. It may be possible that the more complex target performances (COD, LS) benefit more from a higher training volume and intensity of the elite players than simpler exercises (e.g., SJ).

A limitation of this study is the difference in sample size between the groups (elite vs. amateur), which is due to the limited scheduling availability of the amateur athletes. Unbalanced sample sizes may increase the error in difference calculations, but this is mitigated by appropriate corrections to the calculations (e.g., Roy’s largest root or Hedges’ g instead of Cohen’s d). An additional limitation is the ad hoc sample, which can generally increase the sampling error. However, the data set is valuable because only a few studies have investigated and compared young male soccer players [16,17,18,19,20,21].

## 5. Conclusions

The data from this study show performance differences in performance tests depending on the level of play. The SJ, LS, 505, and IAT may prove useful in talent selection test batteries to separate between competitive levels in youth soccer players. The more complex the performance tests were, the larger the effect sizes of the performance differences were. Therefore, the LS and COD tests should be integrated into test batteries. On the contrary, anthropomorphic factors do not significantly differentiate between the level of play and should therefore not be used for selection.

## Figures and Tables

**Table 1:** Mean coefficients of variation, intraclass correlation coefficients, ninety-five percent confidence intervals of the performance tests.

Tests	ICC (95% CI)	CV (95% CI)
Linear sprint 10 m	0.97 (0.95–0.98)	1.4% (1.1%–1.6%)
Linear sprint 20 m	0.98 (0.97–0.99)	1.2% (0.1%–1.4%)
Squat jump	0.97 (0.95–0.98)	4.4% (3.6%–5.2%)
IAT	0.98 (0.97–0.99)	1.5% (1.0%–2.0%)
505	0.96 (0.94–0.98)	2.9% (2.3%–3.5%)

CV = coefficients of variation; ICC = intraclass correlation coefficients; 95% CI = ninety-five percent confidence intervals.

**Table 2:** Mean, standard deviation, absolute and relative difference, t-value, *p*-value, and effect size within the age group of under 19 years old.

Tests	Elite (*n* = 12) Mean ± SD	Amateur (*n* = 11) Mean ± SD	abs. Mean ∆ (%)	Effect
Height (cm)	179.2 ± 6.7	176.6 ± 10.9	2.6 (1.5%)	0.27
Body mass (kg)	74.0 ± 7.7	67.0 ± 14.6	7.0 (9.5%)	0.59
BMI (%)	23.0 ± 1.3	21.4 ± 4.3	1.6 (7.0%)	0.48
Linear sprint 10 m (s)	1.67 ± 0.06	1.82 ± 0.08	0.15 (9.0%) *	2.03
Linear sprint 20 m (s)	2.89 ± 0.08	3.19 ± 0.17	0.30 (10.4%) *	2.24
Squat jump (cm)	40.45 ± 2.91	34.61 ± 6.21	5.84 (14.4%) *	1.18
IAT (s)	13.66 ± 0.36	17.19 ± 0.61	3.53 (25.8%) *	6.84
505 (s)	2.15 ± 0.06	2.64 ± 0.16	0.49 (22.8%) *	4.18

505 = 505 agility test, IAT = Illinois agility test, cm = centimeter, s = seconds; * = significant (*p* < 0.05), effect size = Hedges’ g.

**Table 3:** Mean, standard deviation, absolute and relative difference, t-value, *p*-value, and effect size within the age group of under 17 years old.

Tests	Elite (*n* = 14) Mean ± SD	Amateur (*n* = 8) Mean ± SD	abs. Mean ∆ (%)	Effect
Height (cm)	175.8 ± 5.0	182.0 ± 7.4	6.20 (3.5%)	1.00
Body mass (kg)	70.8 ± 7.2	70.7 ± 8.4	0.10 (0.1%)	0.02
BMI (%)	22.9 ± 1.8	21.3 ± 1.5	1.60 (7.0%)	0.93
Linear sprint 10 m (s)	1.80 ± 0.05	1.80 ± 0.12	0.00 (0.0%)	0.05
Linear sprint 20 m (s)	3.06 ± 0.09	3.22 ± 0.14	0.16 (5.2%) *	1.40
Squat jump (cm)	34.87 ± 2.64	32.51 ± 4.74	2.36 (6.8%)	0.64
IAT (s)	14.52 ± 0.22	16.23 ± 0.60	1.71 (−11.8%) *	4.12
505 (s)	2.25 ± 0.09	2.53 ± 0.08	0.28 (−12.4%) *	3.01

505 = 505 agility test, IAT = Illinois agility test, cm = centimeter, s = seconds; * = significant (*p* < 0.05), effect = Hedges’ g.

**Table 4:** Mean, standard deviation, absolute and relative difference, t-value, *p*-value, and effect size within the total group.

Tests	Elite (*n* = 26) Mean ± SD	Amateur (*n* = 19) Mean ± SD	abs. Mean ∆ (%)	Effect
Height (cm)	177.3 ± 6.0	178.9 ± 9.7	1.6 (0.9%)	0.20
Body mass (kg)	72.3 ± 7.4	68.5 ± 12.2	3.8 (5.3%)	0.38
BMI (%)	23.0 ± 1.5	21.4 ± 3.4	1.6 (7.0%)	0.63
Linear sprint 10 m (s)	1.74 ± 0.08	1.81 ± 0.10	0.07 (4.0%) *	0.80
Linear sprint 20 m (s)	3.98 ± 0.12	3.20 ± 0.16	0.22 (7.4%) *	1.62
Squat jump (cm)	37.45 ± 3.92	33.73 ± 5.60	3.72 (9.9%) *	0.78
IAT (s)	14.13 ± 0.52	16.79 ± 0.77	2.77 (18.8%) *	4.11
505 (s)	2.20 ± 0.09	2.60 ± 0.14	0.40 (18.2%) *	3.40

505 = 505 agility test, IAT = Illinois agility test, cm = centimeter, s = seconds; * = significant (*p* < 0.05), effect = Hedges’ g.

## Data Availability

The data is not yet publicly available.
